# Corporate Social Responsibility and Employee Outcomes: A Moderated Mediation Model of Organizational Identification and Moral Identity

**DOI:** 10.3389/fpsyg.2017.01906

**Published:** 2017-11-01

**Authors:** Wei Wang, Ying Fu, Huiqing Qiu, James H. Moore, Zhongming Wang

**Affiliations:** ^1^Department of Psychological and Behavioral Sciences, Zhejiang University, Hangzhou, China; ^2^Qingdao International Airport Group Co., Ltd., Qingdao, China; ^3^School of Management, Zhejiang University, Hangzhou, China

**Keywords:** corporate social responsibility, turnover intention, in-role job performance, helping behavior, organizational identification, moral identity

## Abstract

Corporate social responsibility (CSR) research is not new, but its importance to today’s socially conscious market environment is even more evident in recent years. This study moves beyond CSR as simply the socially responsible actions and policies of organizations and focuses on the complex psychology of CSR as it relates to individuals within the organization. Given CSR can positively affect both the individuals within the organization and the organization itself, better understanding and leveraging the mechanisms and conditions of CSR that facilitate desired employee outcomes is crucial for organizational performance. However, scholars lack consensus in determining a theoretical framework for understanding how and under what conditions CSR will make an impact on employees and ultimately organizational performance. This study adds clarity by exploring the effect of perceived CSR on a more comprehensive set of employees’ attitudinal and behavioral reactions (i.e., turnover intention, in-role job performance, and helping behavior) via the mediating mechanism of organizational identification and the moderating condition of moral identity. Hypotheses were derived using social identity theory. Results were based on data obtained from 340 Chinese manufacturing employee-supervisor dyads. This study found that employees’ perceived CSR had an indirect relationship via organizational identification with each of the variables: (1) turnover intention, (2) in-role job performance, and (3) helping behavior. Specifically, the negative relationship between perceived CSR and turnover intention was stronger when employees had higher moral identity and the positive relationship between perceived CSR and in-role job performance and helping behavior was amplified by moral identity. Our findings show how the mediating mechanism of organizational identity and the moderating condition of moral identity work together to improve organizational effectiveness. The findings reveal several ways in which organizations can strategically focus their CSR and human resource efforts, such as applying this model and focusing on moral identity as a key indicator when evaluating employees.

## Introduction

In today’s socially conscious market environment, corporate social responsibility (CSR) has become an increasingly important topic among organizations (Serenko and Bontis, 2009; Wagner et al., 2009; Du et al., 2010) and thus has become an important area of study for scholars in strategy and management (Aguinis, 2011; Rupp et al., 2011). Broadly defined as “context-specific organizational actions and policies that take into account stakeholders’ expectations and the triple bottom line of economic, social, and environmental performance" (Aguinis, 2011, p. 855), CSR 25 years ago was mostly confined to observance of environmental legislation for some organizations and corporate philanthropy for others, but today’s picture is far more complex (McPherson, 2012).

This study moves beyond simple definitions of CSR as just the actions and policies of organizations and focuses on the complex psychology of CSR as it relates to individuals within the organization, for such actions and policies are implemented and influenced by the individuals of the organizations. We begin by explaining our view of CSR from an organizational psychology perspective, which we believe is an increasingly essential theoretical basis for CSR research.

Based on existing theories and empirical evidence, scholars have tried to address many important research questions surrounding CSR by exploring predictors and outcomes from different levels (Waldman et al., 2006; Kolodinsky et al., 2010; Aguinis and Glavas, 2012). According to a review by Aguinis and Glavas (2012), CSR was primarily studied at the macro level (i.e., institutional- or organizational-level) compared to the micro-level (i.e., individual-level). For example, from 1970 to 2011, there were only eight studies specializing in individual-level CSR phenomena, garnering a mere 4 percent of all the received articles from that time span. However, based on our review this number has increased sharply from 2012 to April 2016, with more than 30 studies focusing on the individual-level or micro perspective of CSR being published in mainstream journals. This study’s micro-level perspective considers how individuals perceive and react to CSR policies and actions of the organization (Jones, 2010; Lin et al., 2010; D’Aprile and Talò, 2015), and dives deeper into the underlying mechanisms through which CSR leads to specific outcomes such as individuals’ attitudes and behaviors.

Previous research shows that there are positive effects on individuals who work or intend to work for organizations engaged in CSR activities (Peterson, 2004a; Valentine and Fleischman, 2008; Glavas and Piderit, 2009). Understanding the processes and underlying mechanisms linking CSR with employee outcomes is vital because it allows organizations to create specific interventions to best leverage CSR for positive effects on employees and organizational performance (Aguinis and Glavas, 2012; De Roeck and Maon, 2016).

Scholars have made efforts to determine a theoretical framework for understanding how and under what conditions CSR will make an impact on employees and ultimately organizational performance (Rupp et al., 2013; De Roeck et al., 2014; Farooq et al., 2016). For example, previous studies showed that organizational identification mediates the relationship between CSR and employee outcomes (De Roeck et al., 2014; Farooq et al., 2016). Additionally, Rupp et al. (2013) found that moral identity positively moderates the relationship between CSR and organizational citizenship behavior. However, recent research still does not explain why employees with high moral identity respond better to CSR and how that results in better employee outcomes. This study attempts to close this gap by building a moderated mediation model that that better explains the mechanism through which employees with high moral identity respond better to CSR and consequently obtain greater organizational identification that leads to better outcomes.

We use organizational identification as the mediating mechanism and moral identity as the moderating condition for several reasons. First, research has suggested that CSR initiatives could also generate employee-company identification (Jones, 2010; De Roeck et al., 2014). Second, previous research has found that the relationship between perceived CSR and organizational identification is not without other influencing elements, such as moral identity, the degree to which morality is central to an individual’s identity (Hardy and Carlo, 2005). Third, given that identification is a cognitive construct reflecting the extent to which the organization is incorporated into self-conceptualization, organizational identification is seen as contingent upon factors such as perceived similarity and a shared moral sense with the organization (Van Knippenberg and Sleebos, 2006). Therefore, the combination of an individual’s moral sense (i.e., moral identity) and an organization’s CSR initiatives seem to serve as a precursor to self-categorization as a member of the organization. Drawing on social identity theory (Ashforth and Mael, 1989), this study proposes that employees’ moral identity and perceived CSR will influence organizational identification, and in turn the attitudinal and behavioral outcomes. More specifically, we show how the mediating mechanism of organizational identity and the moderating condition of moral identity work together to improve organizational effectiveness.

In line with the framework of organizational citizenship behavior (OCB), we choose turnover intention, in-role job performance, and helping behavior as three indicators of employee outcomes in this study because they represent a more comprehensive set of employees’ attitudes toward the organization, employees’ behaviors toward the organization, and employees’ behaviors toward their coworkers, respectively. Two of these three employee outcomes have been used individually in previous studies but never have all three been combined with perceived CSR and organizational identification mediation. Thus, this new model not only confirms relations of previous less complicated models, but also provides a new way in which to view the effects that CSR has on internal stakeholders (i.e., employees), the process (i.e., mediating mechanism), and the condition (i.e., moderating mechanism) of CSR.

Finally, this study extends previous research in at least three ways. First, this study furthers the research scope of CSR by focusing on individual-level aspects that specifically address employees’ perceptions and the underlying mechanisms and conditions of CSR that lead to employee outcomes. Although never previously studied together, we specifically show how the mediating mechanism of organizational identity and the moderating condition of moral identity work together to improve organizational effectiveness. Rupp et al. (2013) already tested the moderating role of moral identity between CSR and OCB, but this study aim to provide a more specific and inclusive model that reveals the mechanism between CSR and employee outcomes, in which moral identity has its moderating effect on those employee outcomes through organizational identification. Second, given the fragmented evidence of employee outcomes in previous research, this study provides a more comprehensive view of both attitudinal and behavioral employee outcomes and helps pave the way for examining CSR driven employee outcomes in a more systematic fashion. Third, this study has practical significance as it addresses the challenges of finding effective ways to utilize CSR, such as in evaluating individuals under consideration for hire or advancement, and explores a more comprehensive view of how CSR can be utilized to improve employee outcomes.

### Perceived CSR and Employee Outcomes

Scholars have suggested that how employees perceive their organization’s CSR will influence their attitudes and behaviors to support the organization in accomplishing its social and economic goals (El Akremi et al., 2015; De Roeck and Maon, 2016). Existing literature mainly examines relationships between perceived CSR and different aspects of employee outcomes, such as job satisfaction (Valentine and Fleischman, 2008; De Roeck et al., 2014), organizational commitment (Turker, 2009; Stites and Michael, 2011), and organizational citizenship behavior (Rupp et al., 2013; Lee and Kim, 2015). However, only a few studies explore employee outcomes in a more systematic way, containing both attitudinal and behavioral outcomes of employees that potentially bring a more comprehensive understanding of CSR’s impact on employees (Jones, 2010; Kroh, 2014). Following this research approach, we selected three independent but inherently related employee outcomes as a systematic set of employee outcomes that could result from an organization’s CSR initiatives: turnover intention, in-role job performance, and helping behavior. These three outcomes represent employees’ attitudes toward the organization, employees’ behaviors toward the organization, and employees’ behaviors toward their coworkers, respectively.

The three chosen outcomes have origins in organizational citizenship behavior (OCB), which is a commonly studied outcome in evaluating company policies (De Gilder et al., 2005), especially those related to CSR. As the dimensions of OCB are generally arranged in three categories: favorable attitudes toward organization (e.g., loyalty), favorable behaviors toward organization (e.g., individual initiative), and favorable behaviors toward coworkers (e.g., helping behavior) (Coyle-Shapiro, 2002; Jones, 2010), we followed a similar approach in selecting the three outcomes for this study. However, we went further in selecting factors that are beyond OCB-linked variables. We chose turnover intention and in-role job performance to separately represent attitudes and behaviors toward the organization, rather than those corresponding variables of OCB (e.g., OCB loyalty, individual initiative, etc.), which have already been studied quite comprehensively (Hansen et al., 2011; Rupp et al., 2013; Lee and Kim, 2015). We have done this in part in order to bring employee outcomes closer to organizational performance (Jones, 2010). As for the category of favorable behaviors toward coworkers, we extracted helping behavior from OCB as a selected employee outcome because at its core it represents the essence of CSR and is an appropriate measure of employees’ behavior toward the organization. Surprisingly, it has never been studied in relation to CSR by itself without the other accompanying variables of OCB. For the above purposes, we included measures of these three outcome variables which are organization-oriented and employee-associated.

In defining these three outcomes, we begin with turnover intention, which refers to the extent to which an employee intends to withdraw permanently from the job (Cascio, 1982). When employees perceive their organization as socially responsible, they will be more likely to keep their employment relationships with the organization, resulting in reduced turnover intentions (Hansen et al., 2011). Evidence shows that there is a negative relationship between perceived CSR and turnover intention (Kroh, 2014; Du et al., 2015). This study seeks to confirm this negative relationship along with the other outcomes through the addition of organizational identification.

In-role job performance refers to activities required by employee’s formal job description (Janssen and Van Yperen, 2004). Helping behavior refers to voluntary actions to help or benefit coworkers or others, such as sharing, comforting, rescuing, and helping (Organ, 1988). In-role job performance and helping behavior are also related to employees’ perceived CSR. Based on the assumption that the ethical stance within CSR initiatives and actions can enhance employees’ attachment to the organization (Brammer et al., 2007; Valentine and Fleischman, 2008), researchers have found that CSR initiatives have a positive impact on employees’ organizational commitment (Brammer et al., 2007; Turker, 2009; Stites and Michael, 2011), which can further influence in-role job performance (Rhoades et al., 2001; Eisenberger et al., 2010; Vlachos et al., 2014) and helping behavior (Choi, 2006; Shin et al., 2014). To summarize, turnover intention reflects attitudes toward the organization, in-role job performance reflects behaviors at work, and helping behavior reflects the interpersonal behaviors among coworkers. These three variables are inherently related and support each other. This study uses the combination of these measures because together they provide an all-around description of CSR’s impacts on employees. Thus, we postulate in a new combined fashion that perceived CSR facilitates employees’ in-role job performance as well as helping behaviors toward their coworkers.

*Hypothesis 1:* Employees’ perceived CSR will negatively influence their turnover intention (H1a), and positively influence in-role job performance (H1b), as well as helping behavior (H1c).

### The Mediating Role of Organizational Identification

Previous research has shown that employees’ positive perceptions of the external and internal image of their organization’s CSR efforts leads to stronger organizational identification (Carmeli et al., 2007; Jones, 2010), because employees more positively identify themselves and perceive oneness with organizations that they consider as socially responsible (Turban and Greening, 1997). Organizational identification, or the bonding of the organization and self as one (Dutton et al., 1994), can provide crucial implications for understanding the employee-organization relationship, as it has been predicted to positively relate to numerous favorable employee outcomes, such as OCBs (Bartel, 2001; Dukerich et al., 2002), job satisfaction (Van Dick et al., 2004), and job involvement (Riketta, 2005).

In accordance with the stakeholder perspective (Freeman, 1984; Barnett, 2005), the strength of the stakeholder-organization relationship has a direct impact on stakeholders’ attitudes and behaviors (Waddock and Smith, 2000; Bhattacharya et al., 2009). As the most important internal stakeholders (McWilliams and Siegel, 2001), employees who strongly identify with their organization are more likely to show favorable attitudes and behaviors (Dutton and Dukerich, 1991; Kramer, 1993). Given that employees’ positive perceptions of CSR initiatives promote their organization identification, the favorable perceptions possessed by employees should further facilitate their responses associated with organizational identification. In line with this view, this study uses a social identity perspective and proposes that the relationship between perceived CSR and employee outcomes is mediated by organizational identification. Specifically, when there is organizational identification and employees positively perceive CSR, not only is an enduring relationship built with the organization, but their needs for self-esteem are satisfied (Hogg and Terry, 2001). This results in favorable attitudes and behaviors at work (Van Dick et al., 2006; Bhattacharya et al., 2009).

Previous studies have explored the linkage between perceived CSR and employees’ reactions and the mediating role of organizational identification. For example, perceived CSR promotes employees’ organizational identification which further results in enhanced employee outcomes, such as job performance (Carmeli et al., 2007), intent to stay (Jones, 2010), loyalty (Jones, 2010), commitment (Kim et al., 2010), and job satisfaction (De Roeck et al., 2014). However, this study investigates the mechanism underlying the relationship between perceived CSR and the selected set of employee outcomes, which has not been systematically studied in terms of organizational identification as a mediator. Thus, we propose that the mediating role of organizational identification has three effects on the relationship between perceived CSR and the three outcomes (turnover intention, in-role job performance, and helping behavior).

First, we predict that perceived CSR negatively relates to turnover intention through organizational identification because as employees develop positive self-concepts from their organization, they are motivated to maintain their association with the organization and be consistent with their organization’s values. Evidence also shows that organizational identification negatively relates to turnover intention (Mael and Ashforth, 1995; Wan-Huggins et al., 1998; Van Knippenberg and Van Schie, 2000; Cole and Bruch, 2006).

Second, we postulate that positively perceived CSR enhances in-role job performance via organizational identification because when organizational identification is strong, employees’ positive perceptions of their organization’s CSR will promote their perceived oneness with the organization. For example, stronger organizational identification motivates employees to contribute to behaviors that benefit organizational goals (Haslam and Ellemers, 2005), such as in-role behaviors required by formal job descriptions (Van Knippenberg, 2001). Thus, employees with high organizational identification tend to devote efforts for collective goals by performing well in their individual jobs, leading to enhanced in-role job performance (Van Knippenberg, 2001; Haslam and Ellemers, 2005; Eisenberger et al., 2010).

Third, we propose that perceived CSR relates to helping behavior through organizational identification because employees who have positive CSR perceptions become more identified with their organization and are more likely to go beyond their job requirements by extra-role helping behavior (Ellemers et al., 2004; Upham, 2006). Employees who develop higher levels of organizational identification are more likely to help their coworkers (Bartels et al., 2010). In addition, Shen and Benson (2016) found support that organizational-level CSR initiatives positively relate to employees’ ex-role helping behavior via organizational identification. Thus, this study proposes the following hypothesis regarding organizational identification:

*Hypothesis 2:* Organizational identification mediates the relationships between perceived CSR and turnover intention (H2a), in-role job performance (H2b), and helping behavior (H2c).

### The Moderating Role of Moral Identity

The relationship between perceived organizational initiatives and employees’ reactions is influenced by individual differences, such as personality (Barrick et al., 2013; Colbert et al., 2014), and cognitive style (Orpen, 1994; Eisenberger et al., 2001). We find a similar relationship between CSR initiatives and employee outcomes. In recent years, a few researchers have addressed the potential influence of individual traits on the relationships between perceived CSR and employee outcomes (Jones, 2010; Rupp et al., 2013). Since CSR initiatives reflect an organization’s ethical stance (Kotler and Lee, 2005), how employees perceive and react to these initiatives is likely to be influenced by their individual traits, especially those morality-related, such as moral identity (Liao and Rupp, 2005; Colquitt et al., 2006; Rupp and Bell, 2010). Moral identity describes individual differences in moral characteristics given that individuals can differ in terms of the degree to which being moral is central to their self-concepts (Aquino and Reed, 2002).

The moderating role of moral identity in organizational situations has been researched previously, such as moral identity as a moderator of the effect of organizational injustice on counterproductive work behavior (Wu M. et al., 2014) and as a moderator between supervisor abuse of customers and employee organizational deviance (Greenbaum et al., 2013). Given the evidence that moral identity often moderates the relationship between organizational initiatives and employees’ responses, we also propose that it will moderate the relationship of an organization’s CSR initiatives and employees’ organizational identification.

Moral identity represents knowledge about one’s self-definition in relation to some moral traits (Aquino and Reed, 2002), and can be taken as a source of personal intrinsic motivation (He and Pham, 2014). For individuals with high moral identity, moral schemas are “chronically available, readily primed, and easily activated for information processing” (Lapsley and Lasky, 2001, p. 347). More specifically, people with stronger moral identity are more likely to activate moral identity-based knowledge to manage their behaviors (Aquino et al., 2009). In contrast, people with low moral identity tend to care relatively less about ethics or morality and their moral schemas and moral values are not internalized. Moral identity, as a part of social self-schema, directs people to focus more on moral information (Reed et al., 2007). Thus, for those employees with high moral identity, moral values and moral trait associations become more relevant and emphasized when they process information from their organization, especially the moral information embodied by CSR initiatives.

A high-level of moral identity implies a better likelihood of noticing morality-related information, which helps individuals to develop a greater capacity to identify with others based on morality-related variables. Researchers argue that variables used to identify with others can be abstracted to higher order social identities linked to avocational, political, religious, or ethical groups (Deaux et al., 1995). As one of these social identities, moral identity which directs attention to moral information is linked to groups with moral traits. According to self-categorization theory (Turner et al., 1987), individuals assess the match between a social group and themselves and through a process of self-categorization, one can activate the respective social identity by self-categorizing as a group member. Following an identity-based perspective (Tajfel and Turner, 1986; Hogg et al., 2004), individuals assign themselves to social groups that correspond with their attributes and values to fulfill their psychological needs for attribution and sense of being (De Roeck et al., 2014). Typically, within the same group, people’s definitions of what attributes they have in common capture both the similarities and differences from other groups (Böhm et al., 2013). Therefore, combined with self-categorization, employees with high moral identity are more likely to classify themselves into an organization where there is a prominent CSR perception. Moreover, since these employees’ psychological needs are satisfied by self-categorization and since their self-concepts of moral traits are congruent with the organization and its members (De Roeck et al., 2014), they feel a stronger association to the organization and adopt a high level of organizational identification (Ashforth and Mael, 1989). Consequently, whether employees’ moral identity match their perception of an organization’s CSR initiatives, determines whether the employees identify with the organization.

Employees with high moral identity feel greater congruence with organizations where CSR exists. In addition, employees with high organizational identification exhibit better attitudes and behaviors at work (Tyler and Blader, 2003; Van Dick et al., 2006; Marique and Stinglhamber, 2011). Thus, moral identity influences the effect of perceived CSR on organizational identification, which then has an indirect effect on employee outcomes including turnover intention, in-role job performance, and helping behavior as mentioned previously. Therefore, this study proposes the moderating role of moral identity to be as follows:

*Hypothesis 3:* Moral identity will moderate the indirect effect of perceived CSR on turnover intention (H3a), in-role job performance (H3b), and helping behavior (H3c) through organizational identification, and the effect will be stronger when moral identity is high than when it is low.

**FIGURE 1:**
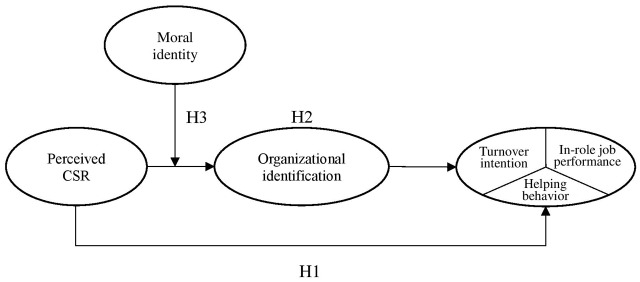
The moderated mediation model.

Based on these hypotheses, we propose a new moderated mediation model that outlines the context where the effects of CSR initiatives on employee outcomes are likely to be influenced by their individual differences related to morality. The theoretical model is schematically represented in **Figure 1**.

## Materials and Methods

### Sample and Procedure

We collected data from ten manufacturing companies located in three different provinces in southern China. We created separate questionnaires for supervisors and their immediate subordinates in order to insure the confidentiality of participants. The subordinate questionnaires were handed out to 375 subordinates, with 108 supervisory questionnaires distributed to their immediate supervisors. Supervisors rated subordinates on their in-role job performance and helping behavior. Subordinates rated themselves on perceived CSR, turnover intention, organizational identification, and moral identity. The number of returned questionnaires was 73 supervisory questionnaires (response rate 68%) and 352 subordinate questionnaires (with response rate 94%).

We removed the unmatched pairs of supervisors and subordinates and obtained a total of 340 supervisor-subordinate dyads as the final sample, of which 43% were women. The mean age of participants was 35.41 (*SD* = 9.39). The average work experience of the participants was 7.91 years (*SD* = 3.02). These participants were engaged in different positions. Most of them (78.7%) worked on the production level, such as quality control and research and development. We focused on employees rather than managers because ordinary employees are more likely to perceive and react to CSR initiatives of a company (Rupp et al., 2006).

### Measures

The present study used two questionnaires for subordinates and their immediate supervisors, respectively. The subordinate version contained demographic variables and scales of perceived CSR, organizational identification, turnover intention, and moral identity. We measured the helping behavior and in-role job performance of subordinates in the supervisory questionnaire that asked respondents to assess their immediate subordinates.

The items were extracted from existing literature and adapted to fit the present study. All measures were translated into Chinese using a procedure of standard translation-back-translation (Reynolds et al., 1993). Likert-type scales were used on all items (1 = ‘*strongly disagree*’ to 5 = ‘*strongly agree*’) to measure the constructs.

#### Perceived CSR

It was measured using a 16-item scale designed by Lin (2010). This scale measures four dimensions: economic, legal, ethical, and discretionary citizenship, each dimension containing four items. Sample items are “My firm provides important job training for employees” (perceived economic citizenship), “My firm always fulfills its obligations of contracts” (perceived legal citizenship), “Fairness toward coworkers and business partners is an integral part of the employee evaluation process in my firm” (perceived ethical citizenship), and “My firm concerned about respecting and protecting the natural environment” (perceived discretionary citizenship). The fit indexes for four first-order factors plus one second-order factor fell within an acceptable range (χ^2^ [100, *n* = 340] = 223.85, *p* < 0.001; TLI = 0.98, CFI = 0.98, RMSEA = 0.06), indicating that these dimensions captured distinctiveness, as well as collective reflectiveness of the overall construct. The Cronbach’s alpha was 0.86.

#### Organizational Identification

It was measured using a 6-item scale developed by Mael and Ashforth (1992). Two sample items are “This organization’s successes are my successes” and “When I talk about this organization, I usually say ‘we’ rather than ‘they’.” The Cronbach’s alpha for this measure was 0.86.

#### Turnover Intention

It was rated by employees using a 5-item scale from Wayne et al. (1997). Two sample items are “I am seriously thinking about quitting my job” and “As soon as I can find a better job, I’ll leave [company name].” The Cronbach’s alpha was 0.85.

#### In-Role Job Performance

It was measured using a 5-item scale from Williams and Anderson (1991). In-role job performance was evaluated by supervisors. Two sample items are “The employee meets formal performance requirements of the job” and “The employee never neglects aspects of the job that he/she is obligated to perform.” The Cronbach’s alpha was 0.89.

#### Helping Behavior

It was measured using a 5-item scale which came from a portion of the scale developed by Coyle-Shapiro (2002) that measures helping behavior. Helping behavior was rated by the supervisors. Two sample items are “Helps others who have heavy workloads” and “Helps others who have been absent.” The Cronbach’s alpha for this measure was 0.90.

#### Moral Identity

It was measured using Aquino and Reed’s (2002) scale. This measure included nine traits, with 10 items and two dimensions (i.e., symbolization and internalization) assessing the importance of the traits. The nine traits were described to the employees as characteristics that may describe a person and were listed as follows: caring, compassionate, fair, friendly, generous, helpful, hardworking, honest, and kind. Participants were asked to imagine a person possessing these traits, and then to evaluate how important having the nine characteristics are to their sense of themselves. For example, two sample items include “The fact that I have these characteristics is communicated to others by my membership in certain organizations” (symbolization) and “It would make me feel good to be a person who has these characteristics” (internalization). The fit indexes for two first-order factors fell within an acceptable range (χ^2^ [34, *n* = 340] = 67.69, *p* < 0.001; TLI = 0.98, CFI = 0.98, RMSEA = 0.05). The Cronbach’s alpha was 0.91.

#### Control Variables

It is included employees’ gender and company tenure. Previous research indicates that these two variables could influence employees’ organizational identification, as well as their attitudes and behaviors (Dutton et al., 1994; Riketta, 2005).

### Analytic Strategy

We ran a confirmatory factor analysis using AMOS 22.0 and adopted 5 general indexes to assess the model fit: χ^2^/*df*, TLI, CFI, RMSEA, and SRMR (Hu and Bentler, 1999). The acceptable cut-off values that we used were less than 2.00 for χ^2^/*df*, more than 0.90 for TLI and CFI, and less than 0.08 for RMSEA and SRMR, which are widely reported and recommended (Hu and Bentler, 1999; Kline, 2011).

We tested the hypothesized model (moderated direct and indirect effects model) using bootstrap methods, applying PROCESS macro (version 2.15), which was first developed by Hayes (2013) and has been iteratively updated until 2016. According to Hayes (2015), the effect of a first-stage moderated mediation is mathematically a linear function of the moderator; and the slope of this function is a product of the coefficient of the XW on M and the coefficient of M on Y^1^, which is also called an INDEX of the moderated mediation. If this index is different from zero, it leads to the expectation that an indirect effect is moderated. We used 5000-sample bootstrapping in this study for all the computations to yield 95% bias corrected confidence intervals. If the confidence interval excludes zero, it leads to the inference that the indirect effect is linearly related to the moderator (Hayes, 2015).

## Results

**Table 1** presents summary statistics and bivarite correlations of the variables.

**Table 1:** Descriptive statistics and intercorrelations of variables.

	Pearson correlations									
	Mean	*SD*	1	2	3	4	5	6	7	8
(1) Gender	0.57	0.50								
(2) Company tenure	7.91	3.02	-0.06							
(3) Perceived CSR	3.04	0.48	-0.07	0.01	**0.86**					
(4) Organizational identification	3.07	0.60	0.03	-0.07	0.41^∗∗^	**0.86**				
(5) Moral identity	3.17	0.68	0.02	0.06	-0.12^∗^	0.16^∗∗^	**0.91**			
(6) Turnover intention	3.08	0.70	0.01	0.03	-0.21^∗∗^	-0.35^∗∗^	-0.01	**0.85**		
(7) In-role job performance	2.91	0.72	0.02	-0.06	0.31^∗∗^	0.43^∗∗^	0.07	-0.50^∗∗^	**0.89**	
(8) Helping behavior	2.97	0.54	0.01	0.01	0.34^∗∗^	0.32^∗∗^	-0.01	-0.49^∗∗^	0.51^∗∗^	**0.90**

*n = 340. Internal consistency coefficients are reported in bold on the diagonal. Gender was recorded as male = 1 and female = 0. ^∗^*p* < 0.05, ^∗∗^*p* < 0.01*.

### Confirmatory Factor Analysis

Using AMOS 22.0, we conducted a confirmatory factor analysis to test whether our hypothesized model captured distinct constructs. The results showed that the hypothesized 6-factor model fit the data in an acceptable way, with χ^2^ [309, *n* = 340] = 465.65, CFI = 0.97, TLI = 0.96, RMSEA = 0.04, and SRMR = 0.05. All of the observed items loaded on their respective latent factors, and the factor loadings were all significant, with a mean of 0.74 indicating that the latent variables had accredited convergent validity. Furthermore, we compared our measurement model to three alternatives: (1) helping behavior and in-role job performance, specified to load on one latent factor, and the other variables loading on their own respective factors, which fit worse than the hypothesized model, with Δχ^2^ [5, *n* = 340] = 584.63, *p* < 0.01; (2) a 4-factor solution with in-role job performance, helping behavior and turnover intention loading on one latent factor, and the other variables loading on their own respective factors, which provided a worse fit than the hypothesized model, with Δχ^2^ [9, *n* = 340] = 929.65, *p* < 0.01; (3) a 2-factor model with the supervisor-rated outcomes loading on one latent factor and employee-rated variables loading on another, providing a significantly worse fit than our measurement model, with Δχ^2^ [14, *n* = 340] = 1759.38, *p* < 0.01. These results indicated that the six constructs captured distinctiveness as expected in the present study.

### The Mediating Role of Organizational Identification

**Table 2** presents the results of a regression analysis of mediating effects (all coefficients are unstandardized). As shown in **Table 2**, the total effects of perceived CSR on turnover intention, in-role job performance, and helping behavior were significantly negative (*b* = –0.31, *p* < 0.001), significantly positive (*b* = 0.46, *p* < 0.001), and significantly positive (*b* = 0.39, *p* < 0.001), thus supporting H1a, H1b, and H1c. **Table 2** also presents the direct effects of perceived CSR on turnover intention, in-role job performance and helping behavior. We also found that the model fit of these mediating effects were acceptable, with turnover intention [*R^2^* = 0.13, *MSE* = 0.44, *F*(2,337) = 25.16, *p* < 0.001], in-role job performance [*R^2^* = 0.20, *MSE* = 0.42, *F*(2,337) = 42.84, *p* < 0.001], and helping behavior [*R^2^* = 0.16, *MSE* = 0.25, *F*(2,337) = 31.74, *p* < 0.001] as dependent variables.

**Table 2:** The regression analysis of mediating effect.

Effect	Variable	*B*	*SE*
Direct effect of X on M	OI	0.51^∗∗∗^	0.06
Direct effect of M on Y	TI	-0.37^∗∗∗^	0.06
	IJP	0.43^∗∗∗^	0.06
	HB	0.20^∗∗∗^	0.05
Total effect of X on Y	TI	-0.31^∗∗∗^	0.08
	IJP	0.46^∗∗∗^	0.08
	HB	0.39^∗∗∗^	0.06
Direct effect of X on Y	TI	-0.12	0.08
	IJP	0.24^∗∗^	0.08
	HB	0.29^∗∗∗^	0.06

*n = 340. OI, organizational identification; HB, helping behavior; IJP, in-role job performance; TI, turnover intention. X, independent variable (perceived CSR); Y, dependent variable (TI, IJP, HB); M, mediator (OI). ^∗∗^*p* < 0.01, ^∗∗∗^*p* < 0.001*.

We adopted bootstrap methods to test the mediating effects by SPSS PROCESS macro (version 2.15), which is concerned with indirect effect (Shrout and Bolger, 2002). We tested the mediating effects by expecting the indirect effects should be non-zero (MacKinnon et al., 1995). We found that the indirect effects of perceived CSR on turnover intention, in-role job performance and helping behavior through organizational identification were -0.19 (95% CI [-0.2873, -0.1105]), 0.22 (95% CI [0.1367, 0.3228]), and 0.10 (95% CI [0.0421, 0.1741]), respectively (see **Table 3**). With all confidence intervals excluding zero, thus H2a, H2b, and H2c were supported.

**Table 3:** The indirect effects of perceived CSR on dependent variables.

Variable	Effect	Boot *SE*	95% CI
Turnover intention	-0.19	0.05	[-0.2873, -0.1105]
In-role job performance	0.22	0.05	[0.1367, 0.3228]
Helping behavior	0.10	0.03	[0.0421, 0.1741]

### The Moderating Role of Moral Identity

We tested the interaction between perceived CSR and moral identity in the first stage, and found that moral identity significantly moderated the relationship between perceived CSR and organizational identification (*b* = 0.23, *p* < 0.001). Conditional indirect effects of perceived CSR on turnover intention, in-role job performance, and helping behavior, computed by PROCESS, were showed in **Table 4**. The conditional effects of the mediator varied at different levels of moral identity (-1 *SD* as Low: -2.49; +1 *SD* as High: 3.86).

**Table 4:** The conditional indirect effects of perceived CSR on dependent variables.

Outcome	Moderator	Effect	*SE*	95% CI
Turnover intention	Low	-0.16	0.04	[-0.2409, -0.0909]
	High	-0.27	0.07	[-0.4201, -0.1497]
In-role job performance	Low	0.18	0.04	[0.1090, 0.2685]
	High	0.31	0.07	[0.1857, 0.4719]
Helping behavior	Low	0.08	0.03	[0.0343, 0.1447]
	High	0.15	0.05	[0.0556, 0.2552]

The indexes of moderated mediation were presented in **Table 5**. The indexes for turnover intention, in-role job performance and helping behavior were -0.08, 0.10, and 0.05, respectively, with all confidence intervals excluding zero. Thus, H3a, H3b and H3c were supported. Moreover, we also tested the moderated mediation model for every dimension of perceived CSR, and found that none of those indexes was significant. We argue that employees’ perceptions of CSR have different aspects (i.e., economic, legal, ethical, and discretionary), and there is no single factor that can reflect all of the perceptions of CSR.

**Table 5:** Index of moderated mediation.

Outcome	Index	*SE*	95% CI
Turnover intention	-0.08	0.04	[-0.1653, -0.0220]
In-role job performance	0.10	0.04	[0.0272, 0.1857]
Helping behavior	0.05	0.02	[0.0090, 0.1004]

**FIGURE 2:**
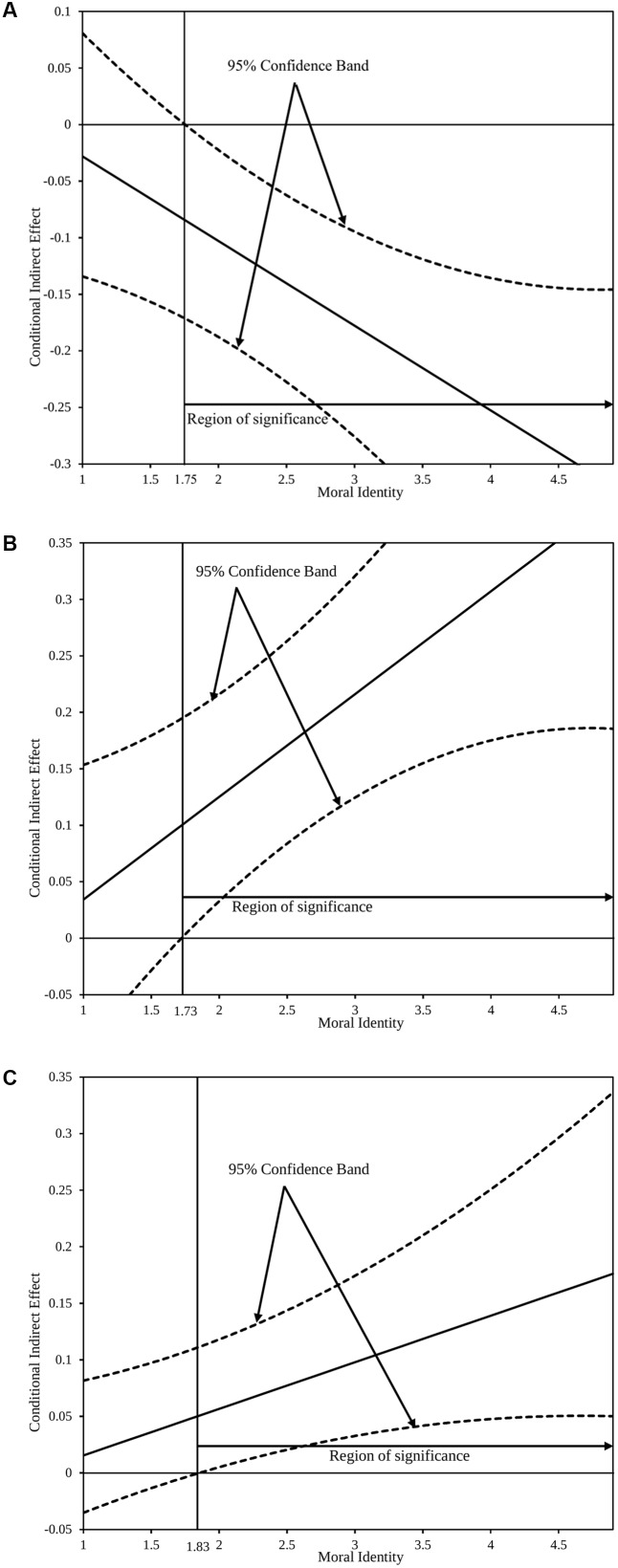
John–Neyman regions of significance for the conditional effects of perceived CSR at values of moral identity. **(A)** Conditional indirect effect of perceived CSR on turnover intention. **(B)** Conditional indirect effect of perceived CSR on in-role job performance. **(C)** Conditional indirect effect of perceived CSR on helping behavior.

To further probe the moderating effect of moral identity, we used the Johnson-Neyman technique (Bauer and Curran, 2005; Preacher et al., 2007; Hayes and Matthes, 2009), which derives the “region of significance” for the conditional indirect effect of perceived CSR in a mathematical way, referring to the values within the range of the moderator where the indirect effects of perceived CSR on those employee outcomes via organizational identification are statistically different from zero. Compared with the more common “pick-a-point” approach, this technique makes a more exhaustive portrait of how moral identity influences the associations between perceived CSR and employee outcomes via organizational identification, especially when moral identity as the moderator is a continuous variable (Pollack et al., 2012). As is shown in **Figure 2**, the vertical lines represent the boundaries of the region of significance, and each pair of dotted curves represents 95% confidence band. **Figure 2A** plots the conditional indirect effect of perceived CSR on turnover intention via moral identity. As can be seen, when moral identity is greater than 1.75 (on a 5-point scale, similarly hereinafter), the indirect effect is significantly negative and different from zero. According to **Figure 2B**, the indirect effect of perceived CSR on in-role job performance through organizational identification is statistically positive and different from zero for any value of moral identity above 1.73. In **Figure 2C**, the indirect effect of perceived CSR on helping behavior via organizational identification is significantly positive when moral identity is greater than 1.83. As long as the value of moral identity falls within the region of significance, the effects of perceived CSR on three dependent variables are significant and amplified by moral identity.

## Discussion

The emerging research on the psychology of CSR has drawn the attention of scholars to employees’ perceptions of and reactions to CSR (Rupp et al., 2013). In this study, we explore the effects of perceived CSR on employee outcomes, the underlying mechanism that may explain these effects, and the moderation of these effects by employees’ moral identity. In short, the results of our empirical test demonstrated that the impacts of perceived CSR on employee outcomes are mediated by employees’ organizational identification, and the indirect effects are bounded by employees’ moral identity. Our findings show that employees perceive their organization’s CSR initiatives based on their own moral identity, and those with higher moral identity respond better to CSR by developing stronger organizational identification, which in turn improves attitudinal and behavioral outcomes. These findings carry several implications for research on organizational theory and social identity theory, as well as CSR initiatives and practice.

### Theoretical Implications

This research extends our knowledge on employees’ reactions toward CSR and its underlying mechanism, making contributions to the psychology of CSR in three notable ways. First, this study reveals the boundary of how perceived CSR influences organizational identification and in turn employee outcomes. Since previous studies show that organizational identification mediates the relationship between perceived CSR and employee outcomes, such as OCBs (Jones, 2010) and job satisfaction (De Roeck et al., 2014), our findings show under what condition employees will get stronger organizational identification and in turn show favorable outcomes when there is a prominent CSR perception. Through lens of social identity theory, we argue that employees with higher moral identity are sensitive toward CSR, as their moral self-definitions are more easily activated when processing moral information (Aquino et al., 2009). If CSR is perceived positively, they feel associated with the organization because of the congruence between their self-concepts of morality and the organization, and are more willing to maintain membership, which triggers stronger organizational identification and ultimately better outcomes.

Second, this study extends the vein of social identity research and enriches the scope of how personal traits (e.g., moral identity) influence organizational behaviors. While Rupp et al. (2013) found that moral identity moderates the effects of perceived CSR on job pursuit intentions and OCBs, our findings suggest that the effects may be more complicated than previously reported by showing that the amplification of moral identity is transmitted to those outcomes through organizational identification. The fact that employee’s moral identity amplifies the effects of CSR initiatives highlights the additional fact that moral-related individual differences could form the base for individuals’ responses to CSR. Since employees with low moral identity might have different moral conceptualizations (Giessner et al., 2015), we speculate that they would use different moral schemas in dealing with CSR initiatives. Whether an employee’s perception of CSR matches his or her moral identity determines whether he or she identifies with the organization, which offers empirical support that social identity theory could help explain why and how employees respond positively or negatively to their organizations’ CSR initiatives. Moreover, the findings also contribute to our understanding of moral identity and its theoretical connections to social identity theory in the field of CSR.

Third, the present study opens the possibility to examine employee outcomes of CSR in a more systematic fashion. As mentioned previously, research has explored the impacts of CSR on employee’s outcomes, such as job satisfaction (Valentine and Fleischman, 2008; De Roeck et al., 2014), organizational commitment (Turker, 2009; Stites and Michael, 2011), and OCBs (Rupp et al., 2013; Lee and Kim, 2015). However, fragmentary evidence cannot portray the overall diagram of employees’ responses toward their perceptions of CSR. The present study chose three variables (i.e., turnover intention, in-role job performance, and helping behavior) to reflect attitudes toward the organization, behaviors toward the organization, and behaviors toward coworkers, respectively. They are inherently connected to each other following the framework of OCB and at the same time they independently depict different aspects of employee outcomes. This synthesis of measures provides a full-scale description of CSR’s impact on employees. The knowledge about how CSR initiatives specifically function within an organization is thus extended by the findings because they reveal comprehensive attitudinal and behavioral aspects of employee outcomes motivated by CSR initiatives, which in turn benefit the organization. Moreover, these three variables of employee outcomes were selected because they reflect employees’ reactions toward CSR directly. In sum, this study suggests a feasible way to reflect CSR’s impacts on employees from a more systematic perspective, and future research could find other potential outcome variables to deepen and widen our understanding of CSR’s impacts.

### Practical Implications

The psychosocial CSR-based model reveals several ways in which organizations can strategically focus their CSR and human resource efforts. First, organizations should take action to sustain the effect of CSR initiatives and commit to more investment in ethical training. Employees’ awareness of their employer’s CSR initiatives will enhance their performance at work as the present study shows, leading to the first suggestion that companies should ensure the acceptance of CSR initiatives by their employees. The results indicate that positive CSR perceptions influence employee outcomes by improving organizational identification. Additionally, as organizational identification is structured on the basis of CSR communications for employees as internal stakeholders (Scott and Lane, 2000; Maignan and Ferrell, 2004), organizations are advised to pay special attention to corporate communication on CSR activities to cultivate employees’ organizational identification.

Moreover, the findings of moral identity in the current study provide evidence of organization’s ethical and moral stance in fostering employees’ organizational identification. The match of ethical and moral cognition helps to establish and maintain the strong relationship between organization and employees, and to better support organizational goals as well (Dutton and Dukerich, 1991; Riketta, 2005). Therefore, it is suggested that organizations should focus more on employees’ occupational ethics, and integrate ethical training into human development strategies. In addition, ethical leadership is also required during CSR initiatives, as it emphasizes treating employees with care, respect and fairness that will lead to high follower identification (Zhu et al., 2015). In addition, responsible leaders more likely encourage employees to identify with the organization (Gond et al., 2011).

Second, as employees are the most important internal stakeholders (McWilliams and Siegel, 2001), organizations should express special concern about the internal side of CSR initiatives. Employees are the main entity that benefits from internal CSR initiatives and will feel obligation to reciprocate similar positive behaviors for the organization once they feel bonded with their organization (Bhattacharya et al., 2009; De Roeck et al., 2014). Accordingly, a high performance of social identity factors is recommended for organizations, such as support systems for employee development and investment. In addition, encouraging employees to participate in CSR activities via moral influence and performance evaluations (Peterson, 2004b; De Gilder et al., 2005) would also be useful (Kim et al., 2010) as it increases their involvement and reinforces their perception and understanding of CSR initiatives. Essentially, learning how to make employees perceive CSR initiatives more fully should become a priority for every organizational leader.

Third, as organizations are considering hiring individuals or evaluating employees for potential advancement or promotion within the organization, applying our model and focusing on the key indicator of moral identity can help to potentially predict future working outcomes. For example, if an employee has a positive perception of CSR coupled with a high-level of moral identity, then the attitudinal and behavioral employee outcomes measured in this study should be magnified via organizational identity. Based on the moderating results of moral identity, we suggest organizations recruit members on the basis of shared values and moral stance in order to maximize the investments organizations make on CSR strategies and initiatives.

### Limitations and Future Directions

The present study has several limitations that future research could overcome, as well as some future directions. First, as this study focused on individual-level only, it would be helpful to explore employees’ responses toward CSR on a multi-level model for future research. Hypotheses could involve cross-level interactions, such as predictors and outcomes from the individual-level with moderators from high-levels (institutional or organizational). Second, since this is a cross-sectional study, the uncertainty of causal relationships exists. Future research may try to employ a longitudinal design to this research area and bring more robust evidence for the mechanisms found in the present study. Third, whereas our data is collected from China, future research can conduct cross-culture comparisons to explore cultural factors that might influence employee’s moral perception and its interactions with CSR initiatives.

Moreover, although helping behaviors and in-role job performance were rated by the subordinates’ immediate supervisors, critical variables that our study mainly focuses on were self-reported by the subordinates themselves. Since self-reported measures may lead to common method variance (Spector, 1994), we followed several procedural remedies to minimize common method variance through the design of the study (see Podsakoff et al., 2003), such as obtaining measures of predictors and criterion variables from different sources, ensuring participants’ confidentiality, and emphasizing that there is no good or bad answer to reduce evaluation apprehension. We measured all the constructs at a single point in time and ran a one-factor model (see Harman, 1967) and its poor fit (χ^2^[324, *n* = 340] = 2615.17, CFI = 0.51, TLI = 0.47, RMSEA = 0.14, and SRMR = 0.12) indicates that no single factor can explain a majority of the variance. Therefore, common method bias should not nullify our findings and future research should also be aware of this potential issue.

Forth, though well-being was not measured in this study, it is reasonable to speculate that these attitudinal and behavioral outcomes measured would be associated with well-being (Rego et al., 2010; Wu J. et al., 2014), which provides the possibility of connecting CSR and well-being by the social identity path based on this study. Since employee’s well-being is important for company’s survival and development (Spreitzer and Porath, 2012), this study enables future research to clarify the various possible interactions of CSR initiatives and employee’s well-being. Finally, this study used organizational identification and moral identity as the mediator and the moderator respectively and reflected employees’ cognition from the individual-level; however, future research could take other factors of employees’ cognitive and psychological foundations into account.

## Ethics Statement

This study was carried out in accordance with the recommendations of Academic Morality Guidelines by the Academic Committee of Zhejiang University with written informed consent from all subjects. All subjects gave written informed consent in accordance with the Declaration of Helsinki. The protocol was approved by the Academic Committee of Zhejiang University.

## Author Contributions

WW, as the first author, mainly proposed the idea and basic model for this research, and collected data for statistics. YF mainly provided her knowledge to develop the hypotheses and build the theoretical model. HQ mainly contributed to the analysis and interpretation of this study. JM mainly contributed to draft the work and revise it. ZW mainly supervised this research and helped revise it. All the authors contributed to ideas, models, data acquisition and analysis, hypotheses developing, writing, revising, and so on. All the authors has approved this research to be published. This research is a result of every author.

## Conflict of Interest Statement

The authors declare that the research was conducted in the absence of any commercial or financial relationships that could be construed as a potential conflict of interest.

## Footnotes

1 X/Y refers to independent/dependent variable, while M/W means mediator/moderator, respectively.
